# Building cohesion in distributed telemedicine teams: findings from the Department of Veterans Affairs National Telestroke Program

**DOI:** 10.1186/s12913-021-06123-x

**Published:** 2021-02-06

**Authors:** Himalaya Patel, Teresa M. Damush, Edward J. Miech, Nicholas A. Rattray, Holly A. Martin, April Savoy, Laurie Plue, Jane Anderson, Sharyl Martini, Glenn D. Graham, Linda S. Williams

**Affiliations:** 1 Center for Health Information and Communication (CIN 13-416), Health Services Research and Development (HSR&D) Service, Richard L. Roudebush VA Medical Center, Indianapolis, Indiana USA; 2 School of Medicine, Indiana University, Indianapolis, Indiana USA; 3 Center for Health Services Research, Regenstrief Institute, Inc., Indianapolis, Indiana USA; 4 VA HSR&D Precision Monitoring for Quality Improvement (PRIS-M) QUERI Center, Indianapolis, Indiana USA; 5 Purdue School of Engineering and Technology, Indiana University–Purdue University Indianapolis, Indianapolis, Indiana USA; 6 VA Office of Specialty Care Services, Neurology, San Francisco, California USA; 7 Department of Neurology, University of California, San Francisco, California USA

**Keywords:** Computer-supported collaborative work, Group cohesion, Social identity, Telemedicine, Virtual communities of practice

## Abstract

**Background:**

As telemedicine adoption increases, so does the importance of building cohesion among physicians in telemedicine teams. For example, in acute telestroke services, stroke specialists provide rapid remote stroke assessment and treatment to patients at hospitals without stroke specialty care. In the National Telestroke Program (NTSP) of the U.S. Department of Veterans Affairs, a virtual (distributed) hub of stroke specialists throughout the country provides 24/7 consultations nationwide. We examined how these specialists adapted to distributed teamwork, and we identified cohesion-related factors in program development and support.

**Methods:**

We studied the virtual hub of stroke specialists employed by the NTSP. Semi-structured, confidential interviews with stroke specialists in the virtual hub were recorded and transcribed. We explored the extent to which these specialists had developed a sense of shared identity and team cohesion, and we identified factors in this development. Using a qualitative approach with constant comparison methods, two researchers coded each interview transcript independently using a shared codebook. We used matrix displays to identify themes, with special attention to team cohesion, communication, trust, and satisfaction.

**Results:**

Of 13 specialists with at least 8 months of NTSP practice, 12 completed interviews; 7 had previously practiced in telestroke programs in other healthcare systems. Interviewees reported high levels of trust and team cohesion, sometimes even more with their virtual colleagues than with co-located colleagues. Factors facilitating perceived team cohesion included a weekly case conference call, a sense of transparency in discussing challenges, engagement in NTSP development tasks, and support from the NTSP leadership. Although lack of in-person contact was associated with lower cohesion, annual in-person NTSP meetings helped mitigate this issue. Despite technical challenges in establishing a new telehealth system within existing national infrastructure, providers reported high levels of satisfaction with the NTSP.

**Conclusion:**

A virtual telestroke hub can provide a sense of team cohesion among stroke specialists at a level comparable with a standard co-located practice. Engaging in transparent discussion of challenging cases, reviewing new clinical evidence, and contributing to program improvements may promote cohesion in distributed telemedicine teams.

## Background

Team cohesion is a key factor in team performance and effectiveness, representing the degree of “belongingness” felt by individual team members, the strength of the shared bond and investment in the overall group, and overall team integration [1, 2]. As healthcare organizations increasingly adopt telehealth platforms for healthcare delivery, building team cohesion in virtual settings takes on new importance to ensure quality care, job satisfaction and workforce retention [3, 4]. The relevance of virtual teams in healthcare has been heightened further by the sharp increase in telehealth encounters during the COVID-19 pandemic. Indeed, McKinsey & Company recently estimated that up to $250 billion in healthcare spending could be delivered in 2020 via telehealth [5].

Telestroke (telemedicine for stroke) connects patients with offsite physicians for stroke evaluation and treatment [6]. Telestroke practices are increasingly considered mature [7], having shown positive effects on various indicators of quality, including cost savings, access to care, efficacy, patient functional outcomes, and patient satisfaction [6, 8, 9]. In the United States, most telestroke programs use a hub-and spoke network model, where stroke specialists employed at a full-service hub facility are consulted by limited-service (often rural) spoke facilities [10–12]. Some programs serve entire states or multistate regions (e.g., The University of Utah Health’s Stroke Center accepts referrals from Utah and six surrounding states).

An alternative network model, the virtual hub (also known as networked, hubless, or distributed), requires no central facility [13–15]. Such hubs are increasingly common, especially in widely distributed healthcare systems (e.g., Victorian [AU] Stroke Telemedicine program [16], Lancashire and Cumbria [GB] Telestroke Network [17], Kaiser Permanente Northern California [US] Stroke EXPRESS [15]). With virtual hub networks, universal access to stroke specialists is increasingly achievable [18].

Virtual hubs also present distinct challenges to building cohesive teams. For specialists, some challenges appear similar to those in physical hubs, including adapting to changes in workload and payment structure [14, 17]. Other challenges increase with program size for virtual-hub specialists. For example, barriers to credentialing and access [19–21] increase with the number of different spoke facilities served. Facing a continuing shortage of neurologists [22], administrators of a successful telestroke program must master recruitment, retention, and continuing education, elements that may have additional challenges in the virtual environment. For example, although recruitment no longer requires a centrally located stroke center, additional effort may be needed for maintaining consistent practice among distributed specialists (e.g., via cross-training [23]). Owing to the difficulty of physical meetings, virtual-hub programs may require great effort in coordinating specialists’ work schedules.

Through its Veterans Health Administration (VHA), the Department of Veterans Affairs (VA) operates the largest integrated healthcare system in the United States. In 2017, the Neurology Program Office in the VA Office of Specialty Care and the Office of Rural Health started a virtual-hub telestroke network, the National Telestroke Program (NTSP) [24]. Although VA uses telemedicine extensively, this is its first nationwide virtual-hub network. Our aims were twofold: 1. To describe the perceptions of the program’s cerebrovascular specialists; and 2. to identify factors in building team cohesion. As little is currently known about the experiences of virtual-hub stroke specialists [25], we were especially interested in identifying training and communication practices for increasing team cohesion and the extent to which stroke specialists located across the US in a virtual service feel like part of a team versus feeling like independent practitioners.

## Methods

We conducted a qualitative, interview-based study of stroke specialists employed by the VA NTSP. We recruited stroke specialists with at least eight months of NTSP employment for semi-structured, confidential interviews. The interviews were collected as part of the ongoing NTSP program evaluation, which was approved as an operational (not research) project. Interviews were audiotaped, transcribed, and analyzed. Analysis of these interviews was approved as expedited research (protocol #1602800879) by the Institutional Review Board at Indiana University and the Research and Development Committee at Richard L. Roudebush VA Medical Center.

### Interviewers

To limit socially desirable responding, we chose interviewers and notetakers (HM, HP, TD, EM, AS) who had no previous relationship with any of the participants [26]. By working in a large, government-run system in a highly regulated industry, we anticipated top-down influences on building the program and on choosing and using communication technology. We practiced reflexivity [27] by using fieldnotes and by avoiding leading phrasing in interviews.

### Population and context

This work was part of a formal evaluation of NTSP, which was deployed and began treating patients in September 2017. By the end of the interviews in May 2018, NTSP included 10 spoke sites and had logged over 150 consultations with local emergency medicine staff. To ensure sufficient experience with the system, we sought specialists with eight months or greater experience in NTSP, excluding program administrators.

NTSP is linked to VHA’s leadership through its Executive Champion, who is also the Deputy Director of Neurology in VHA Specialty Care Services. The program pays specialists per shift worked, not per encounter. For specialists already affiliated with VA, shifts are separate work rather than additional responsibility in their current (non-telestroke) positions. NTSP specialists, regardless of state residence, are credentialed through one facility in VHA and approved to consult on Veterans receiving care at VA facilities in any US state or territory. NTSP is not centrally mandated; medical facility participation is voluntary. Facilities choosing to receive telestroke services from NTSP are both urban and rural and are typically filling a gap in acute stroke care.

### Recruitment, data collection, and data processing

Per the study aims, the interview team (including the lead interviewers) prepared a question-based interview guide (Appendix) on these topics: reasons for initial and continued participation; workload management; methods, facilitators, and barriers to group communication; and affinity with other hub specialists. Opening questions covered background information (e.g., VA appointment percentage, familiarity with anyone in NTSP before joining, and telemedicine practice before NTSP). Later questions were open-ended and concerned team salience; communication methods, facilitators, and barriers; trust in colleagues; and satisfaction with the program.

To identify interview topics, we reviewed literature on healthcare teamwork [28] and virtual communities of practice [29]. Then, we checked the guide for leading phrasing and pretested for face validity (subjective agreement on whether the interview questions sufficiently address our specific aims) by seeking comments from experienced colleagues and NTSP administrators. Using their comments, we revised or removed questions that were likely to have simple, unchanging responses. We kept annotated revisions as documentation.

Interviews were either face-to-face or telephoned, and participation was uncompensated. During interviews, interviewers referred to a printed copy of the guide [30]. Using digital recorders, we audiotaped interviews, which were transcribed by a third-party service. Returned transcripts were stripped of identifiable information and assigned a study identification number.

### Coding and analysis

Five people coded transcripts: the three lead interviewers (HM, HP, TD), one notetaker (EM), and the leader of the NTSP evaluation team (LW, who is a clinical neurologist and health services researcher). We met semimonthly. First, we drafted a codebook using the domains from the interview guide. Then, together, we read two transcripts and revised the codebook until codes were mutually distinct and collectively comprehensive. Between meetings, using qualitative data analysis software (NVivo 12 [31]), two coders independently coded each transcript. In later meetings, our focus moved to finding consensus in paired coding. Then, guided by the framework method [32–34], we identified themes using matrix displays [35, 36], interpreted themes using constant comparison with notes [37], and illustrated themes using direct quotations.

## Results

### Participants and interviews

Interviews were led by HM [6], HP [4], and TD [2]. Each interview lasted between 16 and 48 min (*M* = 33, *SD* = 11). Initial recruitment and the first four interviews were completed at VA’s SimLearn Center (simulation training facility) in Florida, where attendees were learning how to plan and run acute telestroke simulation training sessions. Subsequent recruitment and interviews were done by telephone. Given the program’s small size, we attempted to interview the entire cohort. All 13 eligible specialists who were approached agreed to be interviewed; 12 (92%) were scheduled and interviewed in the second quarter of 2018. The thirteenth was unable to commit to an interview during the study period. All interviewees had completed at least 10 shifts on call, and all interviewees reported completing telestroke consultations (self-estimates ranged between 4 and 20).

Key characteristics of NTSP’s virtual service included its organizational context and membership. NTSP had a flat organization within a larger vertical organization (VA); rather than using intermediaries, NTSP administrators and specialists typically communicated directly with each other and with spoke sites. Eight participants (67%) reported previous practice in telemedicine. Only three participants (25%) knew at least one other NTSP specialist before joining. Four specialists were full-time VA clinical providers, one specialist practiced full time outside VA, and the rest (58%) split their full-time clinical positions between VA and a university affiliate outside of NTSP. Reasons participants gave for joining NTSP included personal interest, professional growth, the flexibility of distributed work, and the ability to help more patients than one could through conventional practice.

### Team implementation facilitators and barriers

We identified facilitators and barriers to team building and classified each as more internal or more external (Table 1). Facilitators consisted of (internal) strengths and (external) opportunities. Facilitators for team building included the perceived trustworthiness of colleagues and administrators. Similarly, barriers to team building consisted of (internal) weaknesses and (external) threats. Weaknesses included not knowing the long-term outcomes of consulted-on patients, which could decrease motivation. The chief threat to team building was the program’s dependence on other offices and vendors for technology integration and management, which could restrict the adoption and use of tools.

**Table 1:** Internal and external facilitators and barriers to team building in NTSP

**Strengths & Facilitators** Interacting with other stroke professionals Being supported by program administration Having trust in fellow specialists Having trust in program’s medical director Flexible scheduling of telestroke shifts Staying current with stroke literature via group	**Weaknesses & Barriers** Unpredictable timing of program changes Little communication of long-term patient outcomes
**Opportunities** Improving onboarding Increasing in-person interaction among specialists Adding video to meetings Expediting call routing as demand increases	**Threats** Depending on other groups for technology integration and management

### Thematic model

**Fig. 1:**
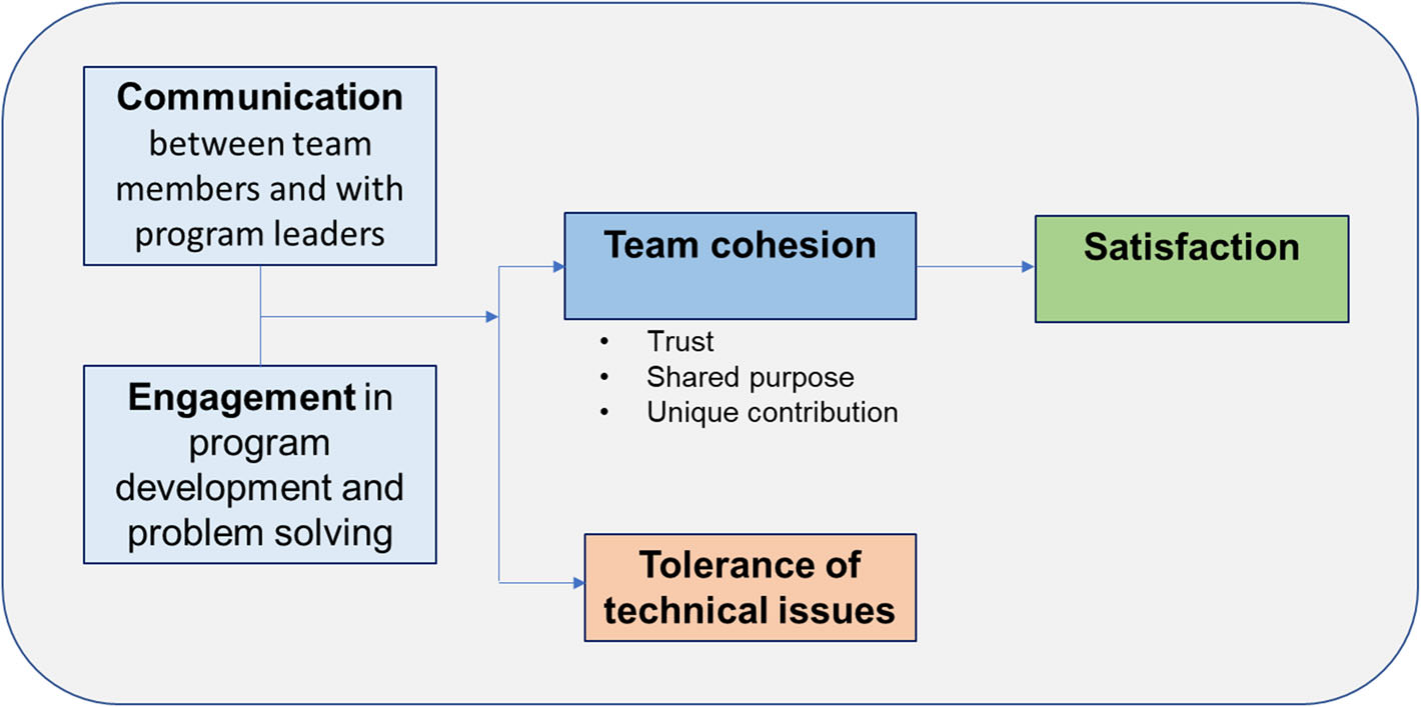
Thematic model

From five related concepts, we identified four themes as phrases [38] and summarized our results in a thematic model (Fig. 1). The model links communication, engagement, team cohesion, and tolerance of technology problems. We propose that team cohesion mediates the link between satisfaction and two other concepts: communication and engagement. Further, we propose that team cohesion moderates the potential negative effect of technology challenges on providers’ satisfaction.

### Communication

Specialists used scheduled and unscheduled communication for complementary goals. Scheduled communication clarified roles. It centered on a weekly case review conference call hosted by NTSP’s clinical leaders. Highlights from this call, attended only by NTSP specialists and administrators, were discussions of challenges, sharing of new research, and reflection on recent notable cases. Between these conference calls, communication consisted of mobile calls, mobile messaging, and electronic mail. Mobile calls were routed to the on-call consultant. Mobile messages used SMS and the mobile chat application WhatsApp (no personally identifiable information or protected health information was communicated). Email services included those provided by VA and other official affiliations. Through these tools, specialists clarified processes and addressed logistics and emergent needs. These tools also connected specialists directly with administrators.

*[My] communications with [NTSP’s medical director] are oftentimes by telephone and it’s, it’s a real plus. I know that if anything significant comes up, I can call her and get a hold of her right away. [P12]*

Conversely, a participant with scheduling conflicts during the weekly calls expressed decreased feeling of team presence.

*Unfortunately, I have my clinic on Monday morning …. That would probably be one of the things that may diminish what I feel in terms of being part of the team. [P11]*

Participants also noticed situations when the communication tool or timing seemed inappropriate for the message (e.g., in urgency).

*[If operational changes are coming, then … ] thoroughly discuss those in the weekly conference calls and then set a time in the future will, where they will be implemented, you know, rather than … sending out an e-mail during the week and indicating the changes expected, you know, right away. [P12]*

### Engagement in problem solving

Specialists took on multiple roles to address the program’s growing needs. Team engagement in program development was a facilitating factor in cohesion. Administrators cultivated team salience by further engaging the specialists in implementation problem solving.

*When I am able to participate with meetings and everything, I just get that feeling that everyone has like fully bought in. [P11]*

Program administrators led by example, and specialists noticed.

*It’s a really ambitious initiative, and obviously, it’s had its kind of challenges as they’ve rolled it out just from a technological and coordination perspective and teaching perspective, but you know. The group that they’ve put together, especially the leadership with [Medical Director] and [Chief], these guys are phenomenal in terms of what their dedication level is to the program. You know. They just really set the tone of just people that are really hard working and are motivated to make this thing as functional and as usable as possible. [P11]*

Administrators fostered an inclusive team culture.

*[NTSP’s medical director], she’s really really good at making sure that everybody is participating and their voice is being heard. I think she really enables the providers to speak up when there’s something that they’re concerned about. I really like that environment. I think that she was very instrumental in building that kind of comfortable cohesive environment, and it really does start with that weekly meeting. That’s probably the best example of it. [P5]*

Engagement in program development included setting up technology for newcomers and aligning schedules. The culture resembled that of a startup company.

*I like it when I see the other providers taking on their own leadership niches because I get to see them at something that they’re good at and can learn something from them. [P1]*

*… the team is very excited about changing the system, improving the system, constantly making it, trying to make it better, and so it’s really exciting working with people that think like that and have that energy, because it gives you energy, gives me energy. [P5]*

### Tolerance of technical issues

Experience with other systems increased specialists’ tolerance of technology barriers. Despite technical challenges, specialists reported high levels of satisfaction with the NTSP. Some of this tolerance was shaped by previous telemedicine practice. But previous practice in telemedicine also served as contrast to NTSP’s problems.

*There are just some times when it’s really difficult to do the right thing for the patient because the technology’s interfering with that. … Like I literally have to pull up a list of my passwords every shift and like make sure they all work because if one of them’s broken then I can’t even complete that shift. [P1]*

Although most technology challenges were perceived as external to the program, some challenges came from software changes within the program. Moving among competing software applications elicited uncertainty.

*That it’s been a little bit challenging in the beginning to get the technology streamlined, especially because we’re juggling a lot of different programs. Initially, we were using one video thing, and then we moved to FaceTime, and then just for me as a non-VA provider, getting on-boarded into the kind of VA construct and getting access to CPRS and VistA [electronic health records] and all of that, that was immensely challenging when patient testing. [P11]*

However, some of the technical challenges resulted in solutions that providers were interested in spreading outside their NTSP practice.

“*Yeah, and actually [Apple FaceTime] that’s something that we are looking to maybe fold into our [academic hospital] system as well based on my experience as a possible backup.” [P11].*

Most of the specialists discussed their competences with telemedicine delivery. They often compared their previous programs with this one. P4 had set up a different neurology telemedicine program, deeming it “frustrating,” and conceded “this [NTSP approach] is the way to do it.”

*Almost all of the people that are doing this [NTSP] are fairly technology savvy, you know what I mean, so like emailing and texting is pretty easy. [P8]*

### Cohesion

Participants found cohesion through the shared experience and unique value proposition of a professional niche. Contributors to cohesion included the weekly case conference call, a sense of transparency in discussing challenges, engagement in program development tasks, and support from program administration. Participants helped each other find solutions to technology problems, which increased the feeling of others’ investment in their own success.

*I think our support system is much better. I think that it’s great that we have our weekly meeting, everybody gets together, we discuss. It’s definitely more a collaborative team. Everybody knows each other. We all talk to each other in our weekly meeting. I think that’s what the VA system is doing better. [P5]*

At times, the level of team cohesion rivaled that of co-located colleagues. For example, one specialist reported a greater frequency of talking with remote colleagues than with co-located ones at the same medical facility and even within a local service.

*We are four neurologists there, you know, in the VA in [City]. One person is hospital coverage … which I was doing before … so I see him, but the other two neurologists I haven’t seen for long. Like one person – [Name] – his wife was texting me. I said I had seen [him] like almost like four months back, you know? So this [NTSP] is more interaction. … So at like certain times you don’t see people in your own facility. [P2]*

To find a sense of belonging within the virtual team, common goals may have helped. Recurring reasons to join across participants were personal growth and professional service.

*I think in general the group feels like the hardest challenges and the most frustrating hurdles have come from outside the group as opposed to within …. You know, technical requirements, security requirements, organizational limitations, or even for example regional culture, like site culture. Like all of those things have been external to our group and so when they come up it feels much more like we’re on the same team trying to find solutions as opposed to anything from within the group causing challenges.* [P1]

Participants reported positive effects of membership on their professional identities.

*I think it [being a telestroke provider at VA] gave me back my identity as a stroke specialist … So, for nine months when I first joined the VA, I did not do a lot of acute stroke. So, finding out about this program was a great bonus ... because I really, I wasn’t sure, I was looking for that chance to do patient interaction and not just treating patients on the camera. So, I think I have a good balance now, so, a lot happier. [P9]*

### Satisfaction

Specialists unanimously praised NTSP and expressed high degrees of satisfaction with the program. They actively identified with the program (“we” appeared often in the transcripts). Several of the specialists pointed out that they enjoyed being part of a virtual team and that they were often the only stroke neurologist on staff at their local VA facility. They developed a collective self-efficacy for problem-solving within the VA environment. They felt like part of a special team with talented members, and being part of this team was meaningful to them. They spoke about practicing at VA in a more positive light because of their work in the NTSP and identifying themselves with the VA organization.

Both communication and engagement worked through cohesion to influence satisfaction. Especially between on-call shifts, communication among participants and with program administrators led to sharing of problems and solutions, promoting unity.

*Well, I enjoy the work, and I also enjoy the people, and I have to say that this [NTSP] program runs well, and being a part of this kind of VA program for me was really sort of a breath of fresh air. [P4]*

Helping each other with technology, a contributor to cohesion, may have also moderated the negative effect of technology issues on satisfaction. For example, to access the electronic health records system from their tablet computers, participants found answers together.

*… we were emailed links but there was no interest on the part of the helpdesk, and I'm not talking about the mobile helpdesk and [IT specialist] who is here with us today, but just sort of the general VA helpdesk, so in the end we sort of kind of figured it out ourselves or found someone in our local IT to sort of figure these things out. [P4]*

## Discussion

### Main findings

In this qualitative study of stroke neurologists employed by the VA NTSP in a virtual service, we identified factors related to team cohesion and satisfaction with distributed work. Strategies for community maintenance included national program leadership support, communication channels, both scheduled and open, and the ability to adopt, change, and clarify internal roles. Having a shared purpose facilitated team cohesion and satisfaction by framing challenges as external to the group and overcome together, rather than internal and alone. These reports, plus favorable comparisons between specialists’ virtual colleagues and their physical counterparts, all indicated that high team cohesion is achievable without regular in-person contact among a virtual clinical team.

NTSP’s early development may be explained by some administrative choices, starting with personnel hiring. Specialists were sought both within and outside VA, ensuring different perspectives on potential best practices. Second, functional communication structures were set up: a virtual meeting with weekly contact, at least one in-person meeting throughout the year, and an open mobile telephone line for urgent needs. Finally, specialists were involved in program development and engaged in ongoing problem-solving. These choices, implemented early, fostered a collective ownership of the program and ensured practice barriers were addressed.

NTSP’s specialists built a virtual community of practice together. First, they participated in the weekly calls and other communication structures. Regular interaction and the weekly calls provided psychological safety for sharing complex cases where best practices were not entirely clear, while flexible scheduling promoted individual participation. In response to the invitation to help develop the program, they took initiative in solving problems and appeared to take collective ownership of NTSP. They reported interacting positively with one another in a responsive manner. This active participation by specialists appeared to be associated with reported satisfaction.

### Novelty

Among reports of virtual-hub networks providing specialty care services (e.g. [15, 39]), to our knowledge, this is the first direct study of specialists’ perspectives. Our findings complement previous studies of other telestroke stakeholders about implementation and sustainability, mostly with the staff of spoke sites [40–42] and hospital and program administrators [43]. Our study also focused on computer-mediated communication among telemedicine specialists between encounters. In a scientific statement from the American Heart and the American Stroke Associations on measuring telestroke provider satisfaction, provider burnout was suggested as an outcome for telestroke providers whose telestroke responsibilities were added to their usual clinical duties [44]. NTSP’s stroke specialists are paid either per shifts completed above their predominant clinical duties or as part of their full-time clinical duties at their respective medical facilities. Thus, provider burnout was not in our findings.

### Integration with previous work

Our study integrates well with prior work linking team cohesion in virtual settings with quality of work, job satisfaction and workforce retention. For example, our results align with those from a study of 14 virtual teams that discovered the relationship between individual trust and team cohesion was reciprocal and created a positive feedback loop [2]. Our qualitative findings also build on the quantitative findings of Tan and colleagues, which found team cohesion to be a dynamic process that is an important factor for virtual team performance [45], as well as those of Lin and colleagues in their meta-analysis of 50 studies that identified team cohesion as one of only five factors (along with relationship building, trust, communication, and coordination) significantly associated with virtual team performance and satisfaction [4]. Moreover, Salvatore reported that greater professional autonomy perceived by physicians was associated with greater organizational identity and pro-social organizational behavior [46]. Our study’s virtual team of stroke specialists reported a strong sense of professional autonomy and both organizational (NTSP) and broader VA identity.

Regarding the implementation of virtual telemedicine hubs, our study builds on a recent qualitative study [43] by identifying neurologists’ positions on governance, training, support, and monitoring and feedback. This study also builds on a previous recommendation for participatory program design [17] and call for physician-led virtual teamwork [25] by describing ways in which hub specialists can aid program development.

Regarding virtual communities of medical practice, our study extends a recent finding [47] that knowledge-sharing helps sustain virtual communities of medical practice without requiring rich communication media. This paper also builds on a 2012 review [21], which found that relationships (e.g., between hubs and their spoke sites) matter more than technology sophistication, by showing one kind of relationship that works well. To address a known lack of formal training for acute telestroke [48], our study shows how NTSP’s weekly calls, discussions of the latest evidence, and open communication between peers and program administrators link specialists in different career stages for on-the-job training.

Last, our study adds to research on physicians’ increasing use of secure, consumer-oriented mobile messaging software like WhatsApp [49, 50]. In our study, mobile messaging was associated with team cohesion by fostering participant satisfaction and ease of communication.

### Strengths and limitations

Strengths of this study include its nearly exhaustive sample recruitment, its focus on an important and unique virtual subspecialist population serving across a national healthcare system, and its infrequently studied context of virtual medical practice. Although limitations center on recruiting from only one healthcare system, telemedicine programs in other healthcare systems may share VHA’s facilitators and barriers (e.g., common electronic health records system and cautious information technology policy, respectively). This study was an early snapshot of the NTSP. Later surveys will assess specialists’ satisfaction with program outcomes over time. Nonetheless, NTSP is a large, national virtual telemedicine program in the US, and this evaluation of team cohesion and satisfaction may inform strategies that future virtual telehealth programs can use to promote professional and organizational identities to minimize staff turnover, a costly implementation barrier.

### Application and future research

Our findings support three recommended actions for developing team cohesion in similar programs: supporting secure, direct communication among specialists and administrators, giving specialists meaningful roles in program development, and examining current practices regularly while implementing changes at predictable times. These actions may help virtual teams successfully address the somewhat universal and persistent challenges of personnel onboarding and changing technology. Establishing best practices for acute telestroke hubs may also help other forms of teleneurology (e.g., hospital teleneurology [51]) and, more broadly, other telemedicine virtual hubs. VA’s Office of Rural Health has invested in the infrastructure to support regional telemedicine hubs like the NTSP for the VA healthcare system. Maintaining a distributed professional workforce is one of its identified challenges.

Future research about virtual teams could include natural experiments (e.g., changes to personnel, workload, technology, funding, or administration) and planned ones (e.g., varying the frequency or mediums of meetings; varying the proportion or frequency of in-person meetings). Exploratory work of interest includes examining the associations between team cohesion and patient and program outcomes. Opportunities for between-group comparison could include studies of virtual team development and salience among other medical subspecialties, comparing a virtual with a traditional health service delivery program, or comparing an acute care team (like telestroke) with a scheduled care team (like outpatient teleneurology consultation). In addition to future research, our study can inform the development of telemedicine program infrastructure or hubs including leadership support and team dynamics to foster organizational identity and provider satisfaction.

## Conclusion

We examined how a national telemedicine program successfully developed team cohesion among a virtual team of stroke specialists. These findings may help administrators build team cohesion in virtual teams in other contexts and disciplines.

## Data Availability

The interview guide is included in the Appendix. A deidentified copy of the dataset analyzed during the study is available from the corresponding author on reasonable request.

## Appendix

### Virtual Stroke Specialists NTSP Team Interview Guide

[Introduce self as part of the NTSP Evaluation Team in Indy].

We are doing this interview to better understand your experience, as part of the National Telestroke Program, of being part of a virtual “hub” of Telestroke providers. The arrangement of a virtual hub of stroke specialists, dispersed over a wide geographic area and different home institutions, is unique in the Telestroke literature and experience, and so we want to better understand the characteristics of this virtual team, and what things help or hinder successful virtual team practices.

By participating in this interview, you are providing verbal assent to the interview process. The NTSP Evaluation is a VA quality improvement initiative, not a research project, and this interview is part of understanding how to improve virtual team experiences and function as this project continues to grow. We will transcribe the interviews, but any identifying information will be removed, and you as an individual will not be identified in any reports or discussion in any way. The interview will take about 30 min. Do you have any questions or concerns about the interview?

### Thanks for agreeing to be interviewed! These questions focus around your experiences and practices as part of the virtual team of stroke specialists in the NTSP.

1. How long have you been a part of the telestroke program at the VA?
  - Probe: Have you completed any Telestroke consults yet? About how many?
  - Probe: How many shifts per month do you work as a telestroke hub provider? Do you work during the day or the evening? Do you work on weekends?
  - Probe: Do you do other clinical work for the VA or is Telestroke your only VA clinical effort? If other VA effort, what clinical care do you provide?
  Probe: About how much of your time on a % basis is outside the VA and how much is within the VA?
2. What would you say were the main reasons you decided to become a VA Telestroke provider?
  - Probe: Before joining the telestroke did you know or work with any of the other hub providers? If so, in what context?
3. What about the NTSP had caused you to keep participating in it?
  Probe: How has your experience as a hub neurologist differed from your original expectations? How has it changed during the course of your involvement?
4. Have you participated in a “traditional” telehealth or hub/spoke community of practice before becoming a VA telestroke provider? (traditional: hub providers located in the same geographic location)
  - If so, how is being a VA Telestroke provider like being a Telestroke provider in that other system? How is it different?
  - What advantages, if any, do you think the VA Telestroke system offers you as a provider over the other system? What advantages, if any, does the other system offer compared to the VA NTSP?
5. To what extent, as a VA Telestroke provider, do you feel like a part of a virtual healthcare team, or a virtual group practice?
  - Probe: What’s an example you can share about a time when the telestroke providers worked well together in providing stroke care? What’s an example of a time when more teamwork among the telestroke providers might have been helpful in providing stroke care?
6. What tools or methods do you use to communicate with other hub neurologists?
  - Probe: (for each method mentioned): How often do you use that method? What kind of information do you share via that method? Does that method involve all NTSP hub providers or just some? Does that method involve the NTSP national team or just hub providers?
7. What makes it easy to communicate with other NTSP providers? What makes it difficult?
8. How does telestroke fit in with your other clinical responsibilities?
  - Probe: Did becoming a hub provider require you to change anything about your clinical schedule?
  - Probe: What do other colleagues (VA or non-VA) that are not part of the NTSP feel about your work in this program?
  - Probe: How does being a VA Telestroke provider affect your overall professional identity as a stroke specialist? Do you see yourself primarily as a university provider or a VA provider or both?
9. In your opinion--what does the National telestroke team do to facilitate a team environment (making you feel as though you are part of a team) of providers?
  - Probe: What could they do more of to facilitate a team environment for the hub providers? (Examples if they can’t offer anything: during onboarding, promoting regular communication (in-person, telephone, electronic?), providing other platforms for interactions virtually and in person)
10. To what extent do you feel you can trust your fellow Telestroke providers in the same way you trust a local stroke colleague? Why or why not?
  - Probe: Do you trust their clinical judgements? Why or why not?
  - Probe: Do you trust their communication with you?
  - Probe: Do you trust their communication with the local VA sites?
  Probe: Are there things that the NTSP could do to increase your trust of your virtual colleagues?
11. Overall, how satisfied are you with the NTSP?
  - Probe: What changes in the NTSP would increase your satisfaction as a provider in the virtual hub of stroke neurologists?
12. Has participating in the NTSP influenced your thoughts about practicing in the VA healthcare system?
  - Probe: Any other positive influences on your opinion about working in the VA?
  - Probe: Any negative influences on your opinion about working in the VA?

What else should we know about the National Telestroke Program that hasn’t come up yet in our discussion?

Thanks so much for taking the time to talk with me.

## Abbreviations

VA: United States Department of Veterans Affairs
NTSP: National Telestroke Program
VHA: Veterans Health Administration
EXPRESS: EXpediting the PRocess of Evaluating and Stopping Stroke
SMS: short message service
